# F^18^-FDG PET imaging as a diagnostic tool for immune checkpoint inhibitor–associated acute kidney injury

**DOI:** 10.1172/JCI182275

**Published:** 2024-08-08

**Authors:** Shruti Gupta, Olivia Green-Lingren, Sudhir Bhimaniya, Aleksandra Krokhmal, Heather Jacene, Marlies Ostermann, Sugama Chicklore, Ben Sprangers, Christophe M. Deroose, Sandra M. Herrmann, Sophia L. Wells, Sarah A. Kaunfer, Jessica L. Ortega, Clara García-Carro, Michael Bold, Kevin L. Chen, Meghan E. Sise, Pedram Heidari, Wai Lun Will Pak, Meghan D. Lee, Pazit Beckerman, Yael Eshet, Raymond K. Hsu, Miguel Hernandez Pampaloni, Arash Rashidi, Norbert Avril, Vicki Donley, Zain Mithani, Russ Kuker, Muhammad O Awiwi, Mindy X. Wang, Sujal I. Shah, Michael D. Weintraub, Heiko Schoder, Raad B. Chowdhury, Harish Seethapathy, Kerry L. Reynolds, Maria Jose Soler, Ala Abudayyeh, Ilya Glezerman, David E. Leaf

**Affiliations:** 1 Division of Renal Medicine, Brigham and Women’s Hospital, Boston, Massachusetts, USA.; 2 Adult Survivorship Program, Dana-Farber Cancer Institute, Boston, Massachusetts, USA.; 3 Harvard Medical School, Boston, Massachusetts, USA.; 4 Department of Radiology, Division of Nuclear Medicine and Molecular Imaging, Brigham and Women’s Hospital, Boston, Massachusetts, USA.; 5 Department of Critical Care & Nephrology, King’s College London, Guy’s and St. Thomas’ Hospital, London, United Kingdom.; 6 King’s College London & Guy’s and St. Thomas’ PET Centre, London, United Kingdom.; 7 Biomedical Research Institute, Department of Immunology and Infection, UHasselt, Diepenbeek, Belgium.; 8 Department of Nephrology, Ziekenhuis Oost Limburg, Genk, Belgium.; 9 Nuclear Medicine, University Hospitals Leuven, Leuven, Belgium.; 10 Division of Nephrology and Hypertension, Mayo Clinic, Rochester, Minnesota, USA.; 11 Nephrology Department, San Carlos Clinical University Hospital, Madrid, Spain.; 12 Department of Radiology, Division of Nuclear Medicine, Mayo Clinic, Rochester, Minnesota, USA.; 13 Department of Biostatistics, Harvard T.H. Chan School of Public Health, Boston, Massachusetts, USA.; 14 Department of Medicine, Division of Nephrology, and; 15 Department of Radiology, Massachusetts General Hospital, Boston, Massachusetts, USA.; 16 Renal Service, Memorial Sloan Kettering Cancer Center and Weill Cornell Medical College, New York, New York, USA.; 17 Institute of Nephrology and Hypertension, Sheba Medical Center and Sackler School of Medicine, Tel Aviv University, Tel Hashomer, Tel Aviv, Israel.; 18 Department of Nuclear Imaging, Chaim Sheba Medical Center, Ramat Gan, Israel.; 19 Division of Nephrology and; 20 Department of Radiology and Biomedical Imaging, University of California San Francisco, San Francisco, California, USA.; 21 Division of Nephrology and Hypertension, University Hospital Cleveland Medical Center, Cleveland, Ohio, USA.; 22 Department of Radiology, Nuclear Medicine, University Hospitals, Cleveland, Ohio, USA.; 23 Katz Family Division of Nephrology and Hypertension, Department of Medicine, University of Miami Miller School of Medicine, Miami, Florida, USA.; 24 Department of Radiology, Division of Nuclear Medicine, University of Miami, Miami, Florida, USA.; 25 Division of Diagnostic Imaging, University of Texas Health Science Center at Houston, Houston, Texas, USA.; 26 Division of Internal Medicine, Section of Nephrology, The University of Texas MD Anderson Cancer Center, Houston, Texas, USA.; 27 Department of Pathology, Brigham and Women’s Hospital, Boston, Massachusetts, USA.; 28 Department of Radiology, Division of Abdominal Imaging and Intervention, Brigham and Women’s Hospital, Boston, Massachusetts, USA.; 29 Molecular Imaging and Therapy Service, Memorial Sloan Kettering Cancer Center, New York, New York, USA.; 30 Division of Hematology-Oncology, Massachusetts General Hospital, Boston, Massachusetts, USA.; 31 Vall d’Hebron University Hospital, Vall d’Hebron Institute of Research, CSUR National Unit of Expertise for Complex Glomerular Diseases of Spain, Barcelona, Spain.

**Keywords:** Nephrology, Oncology, Cancer immunotherapy

**To the Editor:** Immune checkpoint inhibitors (ICIs), anticancer agents that enhance antitumor response, can cause autoimmune toxicities, including ICI-associated acute kidney injury (ICI-AKI). The most common histopathologic lesion in patients with ICI-AKI is acute tubulointerstitial nephritis (ATIN); however, a definitive diagnosis of ATIN requires a kidney biopsy (1). This represents a frequently encountered clinical challenge for providers, as AKI is very common among cancer patients, many of whom have contraindications to kidney biopsy (e.g., solitary kidney, therapeutic anticoagulation). Accordingly, noninvasive methods of diagnosing ICI-AKI are urgently needed, as treatment involves glucocorticoids and discontinuation of potentially life-saving immunotherapy.

Case reports and one case series explored the utility of 2-deoxy-2-[^18^F]fluoro-d-glucose positron emission tomography–computed tomography (F^18^-FDG PET-CT) for diagnosing ICI-AKI and reported mixed findings (2, 3); however, these studies did not have clear inclusion and exclusion criteria to carefully phenotype the patients, did not use rigorous techniques to minimize sampling error, and, most importantly, in some cases did not include a control group. We sought to address these key knowledge gaps and define the role of F^18^-FDG PET-CT in diagnosing ICI-AKI.

We used data from a retrospective, multicenter cohort study of 429 patients with ICI-AKI treated at 30 sites across 10 countries (1). Patients were diagnosed with ICI-AKI between 2012 and 2023 and had either biopsy-proven or clinically adjudicated ICI-AKI (Supplemental Table 1; supplemental material available online with this article; https://doi.org/10.1172/JCI182275DS1), specifically ICI-ATIN.

We also assembled two control groups, each consisting of patients with cancer treated at Mass General Brigham (MGB). The first comprised patients with AKI from non-ICI etiologies, and the second comprised patients treated with ICIs who did not have AKI at the time of a follow-up F^18^-FDG PET-CT.

For all three groups, patients were included if they had F^18^-FDG PET-CT scans at baseline and within 14 days of AKI onset (or, for the second control group, a follow-up scan between 90 and 365 days following ICI initiation). Patients were excluded from all three groups if they had genitourinary cancer, lymphomatous infiltration of the kidneys, or received 7 or more days of glucocorticoids prior to the follow-up scan.

Radiologists at each site reviewed the F^18^-FDG PET-CTs. They were unaware of group assignment at the time of review. Five 0.5 cm diameter regions of interest (ROIs) were drawn in the cortex of each kidney, avoiding the collecting system and space-occupying lesions, such as cysts. The ROIs were selected to represent each kidney’s upper, mid, and lower poles. The mean standardized uptake value (SUV_mean_) for each ROI was recorded.

Fifty-three patients were included (9 with ICI-AKI, 24 with AKI from non-ICI causes, and 20 ICI-treated without AKI; Supplemental Figure 1). Baseline characteristics were largely similar among the three groups (Supplemental Table 2), as were F^18^-FDG PET-CT scan technical parameters (Supplemental Table 3).

Detailed characteristics of the 9 ICI-AKI patients are shown in Supplemental Table 4. Three had biopsy-proven ATIN, whereas the remaining 6 had clinically adjudicated ICI-ATIN. All had clinical features supporting a diagnosis of ATIN (Supplemental Table 5). Those with AKI from non-ICI causes had prerenal AKI (*n* = 10), ischemic or septic acute tubular necrosis (*n* = 10), or other AKI etiologies (*n* = 4) (Supplemental Table 6).

Representative images from baseline and follow-up F^18^-FDG PET-CTs from an ICI-AKI patient (no. 1) are shown in Figure 1A. Among those with ICI-AKI, the SUV_mean_ increased by a median of 57.4% (IQR, 40.3 to 119.8) from baseline to follow-up. In contrast, it increased by 8.5% (IQR, 1.4 to 19.9) among patients with AKI from non-ICI causes and decreased by 0.8% (IQR, -16.6 to 5.1) among patients receiving ICIs without AKI (*P* < 0.001; Figure 1B). The increase in SUV_mean_ in patients with ICI-AKI was also greater compared with that of patients with AKI from non-ICI causes when stratified by AKI etiology (Supplemental Figure 2). The AUC for the differentiation of ICI-AKI from the two control groups according to percentage change in SUV_mean_ was 0.97 (95% CI, 0.93 to 1.00) (Figure 1C). In a sensitivity analysis (described in the Supplemental Methods), the AUC was unchanged at 0.97 (95% CI, 0.92 to 1.00).

In the ICI-AKI cohort, there was little intraindividual variability in the ROIs at each time point (Supplemental Figure 3), though overall precision improved monotonically with a greater number of ROIs (Supplemental Figure 4).

We found that patients with ICI-AKI had a considerable increase in SUV_mean_ on F^18^-FDG PET-CT from baseline to the time of AKI compared with two groups of control patients. These findings suggest that, when a baseline F^18^-FDG PET-CT is available, these scans have diagnostic utility in differentiating ICI-AKI from AKI caused by other etiologies and could offer a noninvasive alternative to kidney biopsy.

Though predominantly used for cancer staging and assessing treatment response, F^18^-FDG PET-CTs have also been used to examine autoimmune toxicity resulting from ICIs. Patients with suspected ICI-associated colitis had increased radiotracer uptake in the colon, whereas uptake decreased with treatment with glucocorticoids (4). Another study found that patients with positive F^18^-FDG PET-CTs of the thyroid were more likely to develop ICI-associated hypothyroidism (5).

Fewer data are available on the role of F^18^-FDG PET-CT imaging for ICI-AKI (2, 3). A single-center study examined F^18^-FDG PET-CT scans in 14 patients with ICI-AKI and reported an increase in FDG activity in the renal parenchyma and a decrease in the collecting system (2). However, the study did not exclude patients with genitourinary cancer or those who had received prolonged courses of glucocorticoids prior to the follow-up F^18^-FDG PET-CT scan, nor did the authors compare their findings with controls without ICI-AKI. Further, only a single ROI in the renal cortex was obtained in each patient, which could have resulted in sampling error.

In our study, we compared changes in FDG uptake from baseline to the time of AKI among patients with and without ICI-AKI while also incorporating rigorous inclusion and exclusion criteria. We acknowledge as a limitation that not all patients had biopsy-proven ICI-AKI; however, this reflects clinical practice, where a diagnosis is often made based on established risk factors, clinical features, and an absence of alternative etiologies (1).

In summary, we found that F^18^-FDG PET-CT may be a useful adjunctive test for diagnosing ICI-AKI in patients with baseline imaging available. Larger prospective studies are needed to validate these findings.

## Supplementary Material

Supplemental data

ICMJE disclosure forms

Supporting data values

## Figures and Tables

**Figure 1:**
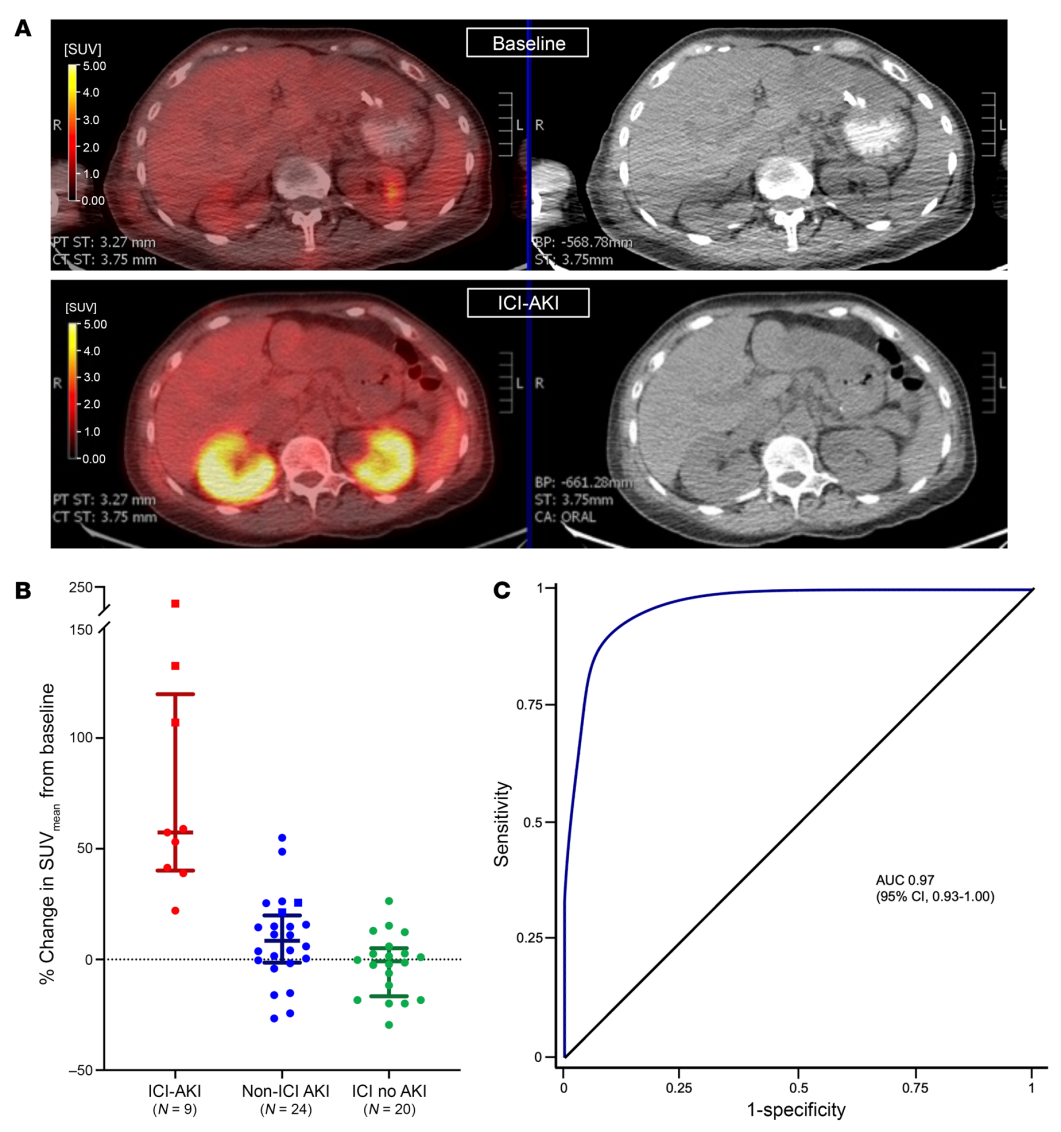
F^18^-FDG PET-CT and ICI-AKI. (**A**) Representative F^18^-FDG PET-CT images at baseline (top panels) and at the time of ICI-AKI (lower panels). (**B**) Percentage change in SUV_mean_ from baseline to the time of AKI among patients with ICI-AKI (red), AKI from other causes (blue), and patients receiving ICI therapy without AKI (green). Biopsy-proven patients are represented by squares, and clinically adjudicated patients with circles. (**C**) ROC curve of percentage change in SUV_mean_ for differentiation of ICI-AKI from AKI from other causes.

## Version 1 08/08/2024

In-Press Preview

## Version 2 09/17/2024

Electronic publication
