# Supplementary material for: F18-FDG PET imaging as a diagnostic tool for immune checkpoint inhibitor–associated acute kidney injury
Source: J Clin Invest. 2024 Aug 8;134(18):e182275. doi: 10.1172/JCI182275 (PMC11405038; doi:10.1172/JCI182275)
Supplement: Supplemental data [file jci-134-182275-s002.pdf]

SUPPLEMENTARY APPENDIX

F^{18}-FDG PET imaging as a diagnostic tool for immune checkpoint inhibitor-associated acute kidney injury

Shruti Gupta,[1,2,3*] Olivia Green-Lingren,[1*] Sudhir Bhimaniya,[3,4*] Aleksandra Krokhmal,[4] Heather Jacene,[3,4] Marlies Ostermann,[5] Sugama Chicklore,[6] Ben Sprangers,[7,8] Christophe M. Deroose,[9] Sandra M. Herrmann,[10] Sophia L. Wells,[1] Sarah A. Kaunfer,[1] Jessica L. Ortega,[1] Clara Garcia Carro,[11] Michael Bold,[12] Kevin L. Chen,[13] Meghan E. Sise,[3,14] Pedram Heidari,[15] Wai Lun Will Pak,[16] Meghan D. Lee,[14] Pazit Beckerman,[17] Yael Eshet,[18] Raymond K. Hsu,[19] Miguel Hernandez Pampaloni,[20] Arash Rashidi,[21] Norbert Avril,[22] Vicki Donley,[21] Zain Mithani,[23] Russ Kuker,[24] Muhammad Awiwi,[25] Mindy Wang,[26] Sujal I. Shah,[27] Michael Weintraub,[4] Heiko Schoder,[28] Raad B. Chowdhury,[1,2,3] Harish S. Seethapathy,[3,14] Kerry Reynolds,[3, 29] Maria Jose Soler,[30] Ala Abudayyeh,[26] Ilya Glezerman,[16] David E. Leaf[1,3]

Table of Contents

Author Affiliations……………………………………………………………………………………………........2
Supplemental Methods…………………………………………………………………………………………………4
Supplemental Tables
Table S1. Criteria for ICI-AKI………………………………………………………...…………………………….…..5
Table S2. Baseline Characteristics ……………………………………………………………………………………..6
Table S3. Characteristics of PET-CT Scan……………………………………………………………………………..7
Table S4. Detailed Characteristics of ICI-AKI…………………………………………………………………….…..8
Table S5. Clinical Features of ICI-AKI Patients.………………………………………………………………………..9
Table S6. Clinical Features of Patients with AKI from non-ICI Etiologies……………………………………….………10
Supplemental Figure
Figure S1. Flowchart of Inclusion…………………………………………………………..…………………………11
Figure S2. Change in SUV_{mean} based on Causes of AKI……………………………………………………………………12
Figure S3. ROIs at Baseline and at ICI-AKI……………………………………………………………….…………13
Figure S4. Precision According to Number of ROIs of Interest..……………………………………………….………14
References………15
Disclosures and Acknowledgements………………………………………………..………………………………..16

SUPPLEMENTAL METHODS

Adjudication of AKI Etiology in Control Patients

Two onconephrologists specializing in the care of patients with cancer and kidney-related issues independently reviewed each of the charts of patients with AKI from non-ICI causes and adjudicated the primary etiology of AKI. They were blinded to the premise of the study, but were specifically asked to comment on their clinical suspicion for ATIN. In the event of a disagreement, a third adjudicator served as a tie-breaker.

Sex as a Biological Variable

Both males and females were involved in the study. Sex was not considered as a biological variable.

Sensitivity Analysis

We conducted a sensitivity analysis similar to the primary analysis except that we excluded patients from the “AKI from non-ICI etiology” control group if they were receiving any of the following medications that are known to cause ATIN, at the time of AKI: non-steroidal anti-inflammatory drugs; trimethoprim-sulfamethoxazole; beta lactam antibiotics; cephalosporin antibiotics; or fluoroquinolone antibiotics.

Statistical Analysis

We calculated an average percent change in SUV_{mean} from baseline to follow-up for each patient. The Kruskal-Wallis test was used to compare the change in SUV_{mean} among the three groups. A receiver operating characteristic curve was generated to evaluate the accuracy of percent change in SUV_{mean} as an indicator of ICI-AKI. Analyses were performed using R version 3.6.3 (R Foundation). All P-values are two-sided, with $P<0.05$ considered statistically significant.

Study Approval

The study was approved with a waiver of informed consent by the institutional review board at each participating site, and by the Mass General Brigham Institutional Review Board for the overall study.

Data Availability

Data from this multicenter study are not publicly available due to contractual restrictions by the participating sites.

Table S1: Criteria for ICI-AKI

**AKI that was directly attributed to the ICI by the treating provider AND either of the following criteria:**	
**Criteria 1:**	Increase in SCr ≥ 100% from baseline OR treatment with KRT
**Criteria 2:**	Increase in SCr ≥50% from baseline AND at least one of the following:<br>1) ATIN on biopsy<br>2) ICI held for at least one cycle due to concern for ICI-AKI<br>3) Treatment with glucocorticoids due to concern for ICI-AKI

Abbreviations: AKI, acute kidney injury; ATIN, acute tubulointerstitial nephritis; ICI, immune checkpoint inhibitor; KRT, kidney replacement therapy; SCr, serum creatinine.

Table S2. Baseline Characteristics

Variable	ICI-AKI (*n*=9)	AKI from non-ICI causes (n=24)	ICI without AKI (n=20)
Age at treatment initiation, yrs, median (IQR)	73 (60-80)	65 (60-74)	67 (61-74)
Male, n (%)	6 (67)	15 (63)	15 (75)
Race, n (%)			
White	8 (89)	20 (83)	20 (100)
Black	0 (0)	1 (4)	0 (0)
Other/Unknown	1 (11)	3 (13)	0 (0)
Comorbidities, n (%)			
Hypertension	3 (33)	13 (54)	10 50)
Diabetes	0 (0)	4 (17)	5 (25)
CHF	0 (0)	2 (8)	0 (0)
COPD	1 (11)	0 (0)	1 (5)
Cirrhosis	0 (0)	0 (0)	0 (0)
Body mass index, median (IQR)	23 (21-28)	27 (24-33)	32 (29-36)
Baseline eGFR,[a] ml/min per 1.73 m			
Median (IQR)	87 (64-101)	61 (60-91)	79 (63-90)
eGFR Categories, n (%)			
≥90	4 (44)	6 (25)	5 (25)
60-89	4 (44)	13 (54)	14 (70)
45-59	0 (0)	5 (21)	1 (5)
<45	1 (11)	0 (0)	0 (0)
AKI Stage,[b] n (%)			
Stage 1	2 (22)	2 (8)	NA
Stage 2	1 (11)	14 (58)	NA
Stage 3	6 (67)	8 (33)	NA
Malignancy, n (%)			
Lung	3 (33)	0 (0)	0 (0)
Melanoma	4 (44)	0 (0)	14 (70)
Lymphoma	2 (22)	15 (62)	0 (0)
Other	0 (0)	9 (38)	6 (30)

Data are shown as median (IQR) and n (%). Abbreviations: ICI, immune checkpoint inhibitor; AKI, acute kidney injury; IQR, interquartile range; CHF, congestive heart failure; COPD, chronic obstructive pulmonary disease; eGFR, estimated glomerular filtration rate; mg/dl, milligram per deciliter; mCi, millicurie; PET-CT, positron emission tomography-computed tomography scan.

[a]Baseline eGFR was defined based on the SCr value closest and prior to ICI initiation, and was calculated based on Chronic Kidney Disease-Epidemiology Collaboration equation without race (1).
[b]AKI was staged according to the Kidney Disease: Improving Global Outcomes criteria (2).

Table S3: Characteristics of PET-CT scan

Variable	ICI-AKI (*n*=9)	AKI from non-ICI causes (n=24)	ICI without AKI (n=20)
Blood glucose, mg/dl, median (IQR)			
Baseline PET-CT scan	95 (86-99)	102 (92-109)	98 (88-115)
Follow-up PET-CT scan	91 (86-108)	102 (93-106)	99 (90-121)
Radiotracer dose, mCi, median (IQR)			
Baseline PET-CT scan	11.7 (9.1-15.9)	14.4 (9.9-15.1)	14.9 (9.9-20.0)
Follow-up PET-CT scan	10.0 (7.9-13.6)	11.0 (10.0-14.9)	10.3 (9.9-19.9)
Uptake time, min, median (IQR)			
Baseline PET-CT scan	61 (59-81)	64 (59-68)	62 (56-72)
Follow-up PET-CT scan	70 (64-75)	59 (54-69)	69 (58-74)
SUV_{mean}, median (IQR)			
Baseline PET-CT scan	1.8 (1.6-2.4)	2.1 (1.9-2.5)	2.3 (2.0-2.5)
Follow-up PET-CT scan	3.2 (2.5-4.3)	2.2 (2.0-2.6)	2.1 (1.9-2,4)

Data are shown as median (IQR) and n (%). Abbreviations: IQR, interquartile range; mg/dl, milligram per deciliter; mCi, millicurie; PET-CT, positron emission tomography-computed tomography scan.

Table S4: Detailed Characteristics of ICI-AKI Patients

Pt #	Age/ Sex	Malignancy	ICI	SCr (mg/dl) BL / Peak / Nadir[a]	Proteinuria (dipstick/ UPCR)	LE / Blood (dipstick)	Extra-renal irAE	SUV_{mean} BL / ICI-AKI (% change)
1	75/F	Non-Hodgkin lymphoma	Nivo	0.80/4.5/0.9	3+/0.74	neg/neg	None	1.6/5.2 (228.4)
2	58/F	Lung adeno	Pem	0.6/2.9/0.6	neg/0.35	3+/neg	None	1.5/3.3 (132.6)
3	82/M	Lung adeno	Durv	1.8/5.9/2.5	neg/1.15	2+/1+	Pneumonitis	2.3/4.7 (107.0)
4	56/F	Melanoma	Ipi and Nivo	0.7/4.8/1.0	neg/0.33	1+/1+	None	1.9/3.0 (59.0)
5	73/M	Melanoma	Ipi and Nivo	0.9/2.0/1.0	neg/NA	neg/1+	Pneumonitis	1.5/2.3 (57.4)
6	78/M	Hodgkin lymphoma	Nivo	0.9/8.4/0.8	2+/NA	3+/neg	None	2.8/4.2 (53.1)
7	61/M	Melanoma	Ipi and Nivo	1.1/1.9/1.5	neg/0.10	neg/neg	Thyroiditis and pneumonitis	1.7/2.4 (41.4)
8	71/M	Lung SCC	Pem	1.2/9.1/1.9	1+/0.64	3+/neg	Colitis	1.7/2.4 (39.1)
9	85/M	Melanoma	Pem	1.3/2.3/1.7	NA	NA/NA	Arthritis	2.3/2.8 (22.1)

Abbreviations: adeno, adenocarcinoma; AKI, acute kidney injury; BL, baseline; Durv, Durvalumab; ICI, immune checkpoint inhibitor; Ipi, Ipilimumab; irAE, immune-related adverse event; mg/dl, milligram per deciliter; LE, leukocyte esterase; NA, not available; Nivo, Nivolumab; Pem, Pembrolizumab; SCC, squamous cell carcinoma; SCr, serum creatinine; SUV, standardized uptake value; UPCR, urine protein creatinine ratio.

[a]Nadir refers to the lowest serum creatinine value achieved within 90 days following AKI onset.

Table S5: Clinical Features of ICI-AKI Patients

Pt #	Biopsy-proven ATIN?	# Weeks between ICI initiation and AKI	Risk factors/clinical features of ICI-AKI				Plausible alternative cause of AKI?	Treated with GC?	Kidney recovery with GC?[d]	% change in SUV_{mean}
			Combination ICI therapy?[a]	Concurrent PPI use?[b]	Prior or concomitant extrarenal irAEs?	Pyuria?[c]				
1	Yes	19	No	No	No	No	No	Yes	Yes	228.4
2	Yes	43	No	No	No	Yes	No	Yes	Yes	132.6
3	Yes	9	No	No	Yes	NA	No	Yes	Yes	107.0
4	No	28	Yes	Yes	No	No	No	Yes	Yes	59.0
5	No	11	Yes	Yes	Yes	No	No	Yes	Yes	57.4
6	No	17	No	No	No	Yes	No	Yes	Yes	53.1
7	No	10	Yes	No	Yes	NA	No	Yes	Yes	41.4
8	No	20	No	No	Yes	Yes	No	Yes	Yes	35.7
9	No	14	No	Yes	Yes	NA	No	Yes	Yes	22.1

Abbreviations: AKI, acute kidney injury; ATIN, acute tubulointerstitial nephritis; GC, glucocorticoids; ICI, immune checkpoint inhibitor; irAE, immune-related adverse event; NA, not available; PPI, proton pump inhibitor; SCr, serum creatinine; SUV, standardized uptake value.

[a]Refers to simultaneous treatment with a CTLA-4 inhibitor and a PD-1 or PD-L1 inhibitor, as this has been shown to be a risk factor for ICI-AKI (3).
[b]Concurrent PPI use has been shown to be a risk factor for ICI-AKI (4).
[c]Pyuria was defined as ≥10 WBCs per high power field.
[d]Kidney recovery was defined as a return of SCr to within 50% of baseline within 90 days of ICI-AKI. Baseline SCr was defined as the closest SCr prior to ICI initiation.

Table S6: Clinical Features of Patients with AKI from non-ICI Etiologies

Pt #	Age/ Sex	Malignancy	Concomittant Medications			SUV_{mean} BL / AKI (% change)	Etiology of AKI / Clinical Setting
			NSAIDs	PPIs	Antibiotics		
1	50/F	Anal SCC	No	No	No	2.8/2.2 (-16.1)	Prerenal: had high ostomy output; AKI responded to IVF.
2	62/F	Mantle cell lymphoma	No	No	TMP/SMX	2.0/2.0 (5.9)	Prerenal: resolved with supportive care; was on TMP/SMX and acyclovir but no suspicion for ATIN.
3	71/F	Breast cancer	Yes	No	No	2.1/2.3 (11.0)	Prerenal: was taking NSAIDs, poor PO intake, and AKI improved quickly after IVF.
4	61/F	Endometrial	No	No	No	2.1/2.5 (14.5)	Ischemic ATN from poor PO intake superimposed on oxalate nephropathy (biopsy-proven).
5	67/F	DLBCL	No	Yes	Meropenem	1.9/2.1 (15.0)	ATN from sepsis due to candidemia and enterococcal bacteremia. Low suspicion for ATIN from meropenem.
6	74/M	Multiple myeloma	No	Yes	TMP/SMX	2.2/2.5 (15.7)	ATN from light chain cast nephropathy, hypovolemia, and hypercalcemia. Had been on TMP/SMX and PPI for ~5 years.
7	55/M	Multiple myeloma	No	Yes	TMP/SMX	2.8/3.2 (21.3)	Prerenal: poor PO intake from mucositis, pneumonia. On PPI for ~7 years.
8	78/F	Waldenstrom's macroglobulinemia	No	Yes	Amoxicillin	1.9/2.4 (25.7)	ATN and glomerular hypoperfusion on kidney biopsy, without evidence of ATIN.
9	60/M	Oropharyngeal SCC	No	No	No	2.8/2.8 (11.3)	Prerenal: IV contrast and poor PO intake on a background of diabetic kidney disease. AKI improved with supportive care.
10	84/M	DLBCL	No	No	No	1.8/1.9 (3.8)	Hydronephrosis, with AKI improving with resolution of obstruction.
11	61/M	PTLD	No	Yes	Vancomycin	2.2/2.2 (1.6)	Prerenal AKI from CNI toxicity (tacrolimus) and febrile neutropenia. Had received vancomycin and cefepime a few days prior but low suspicion for ATIN. Had been on a PPI for ~ 6 years.
12	74/M	DLBCL	No	Yes	Vancomycin<br>Cefepime<br>Tobramycin<br>Metronidazole	2.0/2.0 (4.2)	ATN secondary to septic shock and neutropenic fever.
13	61/F	Non-Hodgkin's lymphoma	No	Yes	TMP/SMX	3.1/3.1 (-0.4)	Prerenal: diarrhea, poor PO intake; improved with IVF. Had been on omeprazole for >3 years.
14	72/M	DLBCL	No	No	Vancomycin<br>Cefepime<br>Metronidazole	2.5/1.8 (-26.6)	ATN from septic shock and cardiogenic shock.
15	50/M	DLBCL	No	Yes	Cefepime	2.4/3.0 (25.4)	Ischemic ATN in the setting of cytokine release syndrome from CAR-T with third spacing. No suspicion for ATIN from PPI or cefepime.
16	64/M	DLBCL	No	No	Levofloxacin<br>Ceftazadime	1.9/1.9 (0.4)	Prerenal: hypercalcemia, concomitant infection. On TMP/SMX prophylaxis but no suspicion for ATIN.
17	76/M	CLL	No	No	Ciprofloxacin<br>Amoxicillin	1.9/1.9 (-4.2)	Ischemic ATN from severe hypovolemia in the setting of hypercalcemia, poor PO intake, lisinopril. Received antibiotics for a UTI after AKI episode.
18	27/M	DLBCL	No	No	Ciprofloxacin	1.9/2.2 (14.8)	Prerenal: improved with IVF. Had also received carboplatin and ifosfamide 7 days prior, but toxic ATN seemed less likely.
19	78/M	DLBCL	No	No	No	1.6/2.2 (48.7)	ATN from TLS in the setting of lymphoma.
20	42/F	DLBCL	No	Yes	TMP/SMX	2.0/2.7 (26.3)	Ischemic ATN in the setting of acute blood loss requiring multiple transfusions. On TMP/SMX prophylaxis but no suspicion for ATIN.
21	67/M	DLBCL	No	No	Ceftazadime<br>Levofloxacin	2.3/2.0 (-15.2)	Sepsis-associated ATN in the setting of neutropenic fever.
22	77/F	CNS lymphoma	No	Yes	No	2.3/2.2 (-1.7)	Toxic ATN: HDMTX received 5 days prior to AKI onset. Had been on a PPI, but no suspicion for ATIN.
23	62/M	DLBCL	No	No	Ceftriaxone	1.3/2.0 (55.0)	Prerenal: poor PO intake, influenza, hypotension. Improved with IVF.
24	66/M	DLBCL	No	Yes	Cephalexin	2.6/2.0 (-24.3)	Ischemic ATN from cardiorenal syndrome, acute blood loss. Had been on a PPI for >10 years, no suspicion for ATIN.

Patients #4 and #8 were biopsy-proven, and the remainder were clinically-adjudicated.

Abbreviations: AKI, acute kidney injury; ATN, acute tubular necrosis; ATIN, acute tubulointerstitial nephritis; BL, baseline; CAR-T, chimeric antigen receptor T-cell therapy; CLL, chronic lymphocytic leukemia; CNI, calcineurin inhibitor; CNS, central nervous system; DLBCL, diffuse large B-cell lymphoma; HDMTX, high-dose methotrexate; IVF, intravenous fluids; NA, not applicable; NSAID, non-steroidal anti-inflammatory drug; PO, per os (oral); PPI, proton pump inhibitor; PTLD, post-transplant lymphoproliferative disorder; TMP/SMX, trimethoprim-sulfamethoxazole; SCC, squamous cell carcinoma; SCr, serum creatinine; SUV, standardized uptake value; TLS, tumor lysis syndrome; UTI, urinary tract infection.

Figure S1. Flowchart of Inclusion

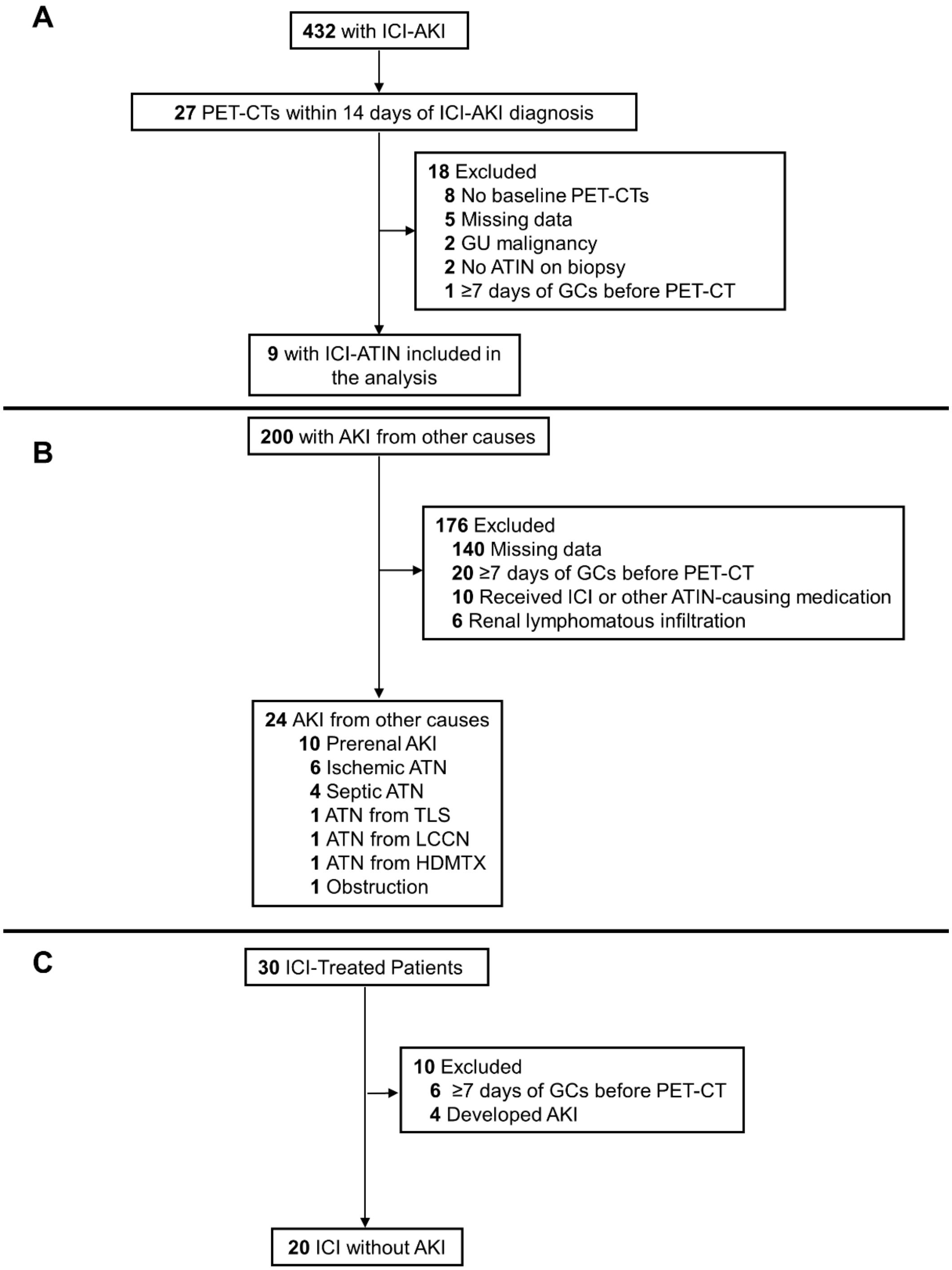

Abbreviations: AKI, acute kidney injury; ATIN, acute tubulointerstitial nephritis; GC, glucocorticoids; GU, genitourinary; HDMTX, high-dose methotrexate; ICI, immune checkpoint inhibitor; LCCN, light chain cast nephropathy; PET-CT, positron emission tomography/computed tomography; TLS, tumor lysis syndrome. The 432 patients with ICI-AKI in panel A were derived from our parent publication (N=429) along with 3 additional patients identified since its publication (4).

Figure S2. Change in SUV_{mean} based on Causes of AKI

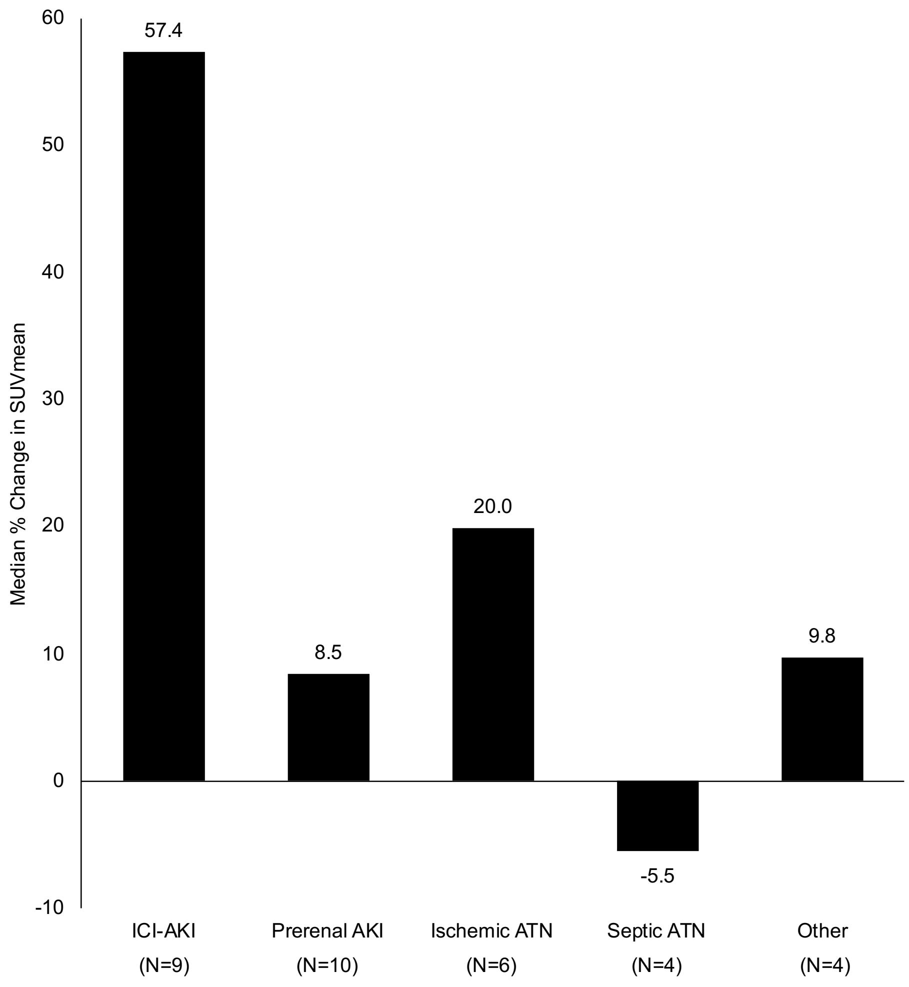

Figure S3. ROIs at Baseline and at ICI-AKI

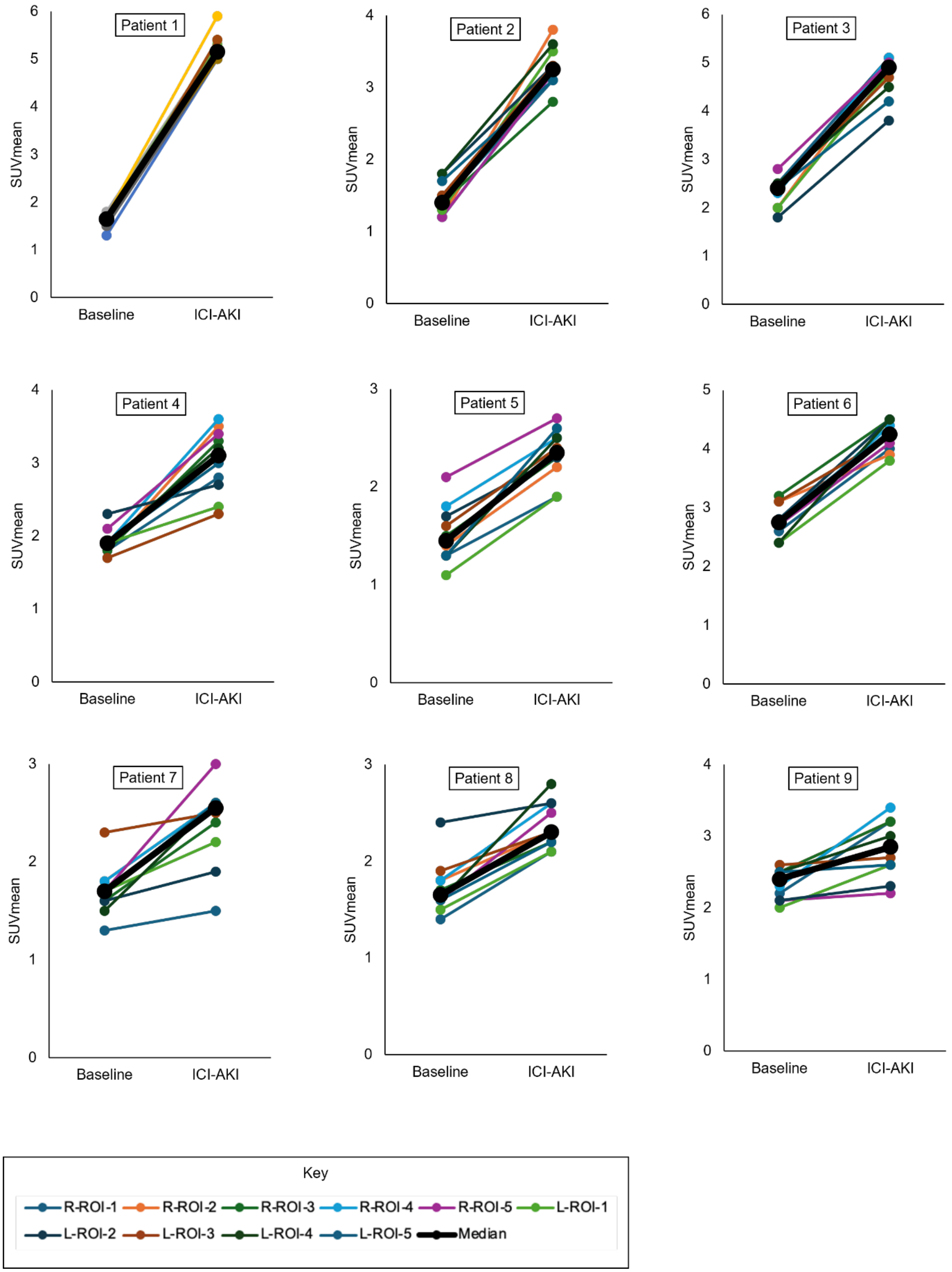

Figure S4. Precision According to Number of ROIs of Interest

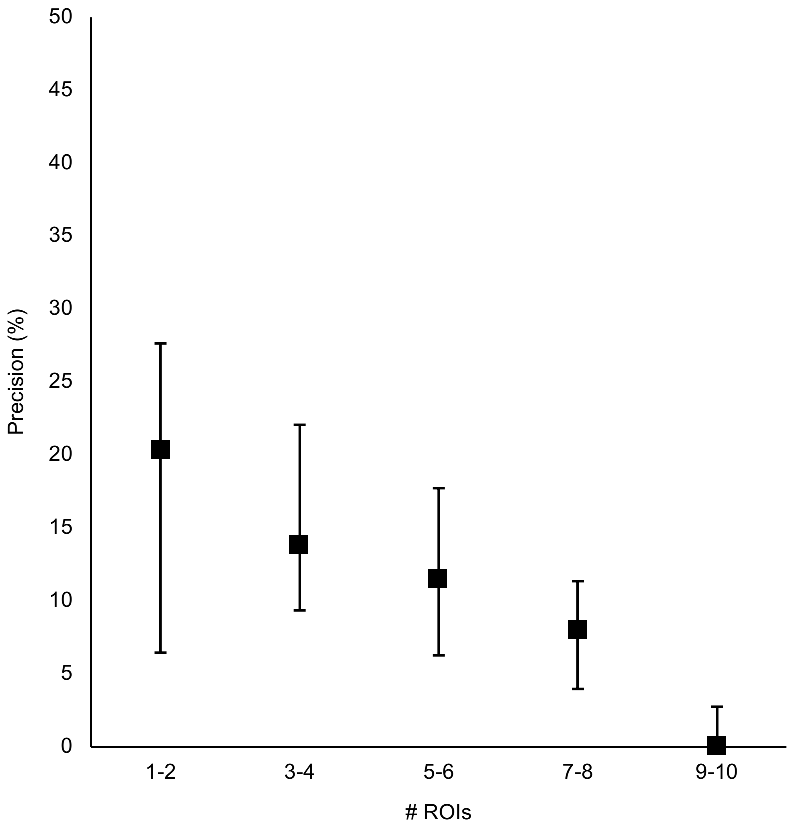

Precision was calculated according to the number of regions of interest (ROIs) as follows: the average change in SUV_{mean} for each patient was calculated according to the number of ROIs (1-2, 3-4, 5-6, 7-8, or 9-10) and compared to the overall SUV_{mean} based on all 10 ROIs. The % difference from the overall SUV_{mean} was calculated for each patient. The median (IQR) % difference was then assessed globally (across all 9 patients) according to number of ROIs (1-2, 3-4, 5-6, 7-8, or 9-10). Precision improved monotonically with a greater number of ROIs.

References

1. Inker LA, Eneanya ND, Coresh J, et al. New creatinine- and cystatin C–based equations to estimate GFR without race. N Engl J Med. 2021;385:1737-1749.
2. Kellum JA, Lameire N, Aspelin P, et al. Kidney disease: Improving global outcomes (KDIGO) acute kidney injury work group. KDIGO clinical practice guideline for acute kidney injury. Kidney Int Suppl. 2012.
3. Cortazar FB, Kibbelaar ZA, Glezerman IG, et al. Clinical Features and Outcomes of Immune Checkpoint Inhibitor-Associated AKI: A Multicenter Study. J Am Soc Nephrol. 2020;31(2):435-446.
4. Gupta S, Short SAP, Sise ME, et al. Acute kidney injury in patients treated with immune checkpoint inhibitors. *J Immunother Cancer*. 2021;9(10):e003467.

FUNDING
This work was not funded by any entity.

ACKNOWLEDGEMENTS
None

AUTHORS' CONTRIBUTIONS
SG, OGL, and DEL designed the study, analyzed the data, and wrote the manuscript. SB analyzed all PET-CTs from patients in the two control groups. All other authors assisted with data collection, analysis, and in critically reviewing and revising the manuscript.

DATA SHARING STATEMENT
The authors are unable to share the raw data publicly. The Coordinating Center at Mass General Brigham has a separate data use agreement (DUA) with each of the other institutions that contributed data to the study. Each DUA has its own specifications to comply with local IRB policies from each of the contributing sites regarding sharing of data. The DUAs do not give the investigators permission to make the data publicly available in any form (with or without identifiers).

CONFLICTS OF INTEREST
S.G. received research support from BTG International, Janssen, AstraZeneca, and the Wong Foundational Grant at Dana-Farber Cancer Institute.
H.A.J received research support from Blue Earth Diagnostics and Lantheus.
M.E.S has received research support from Otsuka, Gilead, Cabaletta, EMD-Serono, and Merck. She has served on Scientific advisory boards or had scientific consulting agreements with Otsuka, and is a data safety monitoring committee member for Alpine Immune Sciences. Her spouse is a consultant for Xbiotix.
M.J.S received research support from Boehringer, Fondo de Investigación Sanitaria-FEDER, Instituto de Salud Carlos III (ISCIII), PI21/01292, RICORS program to RICORS2040 (RD21/0005/0016), ERA-PerMed-JTC 2022 (ONAKI-ICI AC22/00029), and Marató TV3 2021 215/C/2021. She also received research funding from AstraZeneca and honoraria from Novonordisk.
M.O. received research funding from Baxter, bioMerieux and LaJolla Pharma.
I.G's spouse owns Pfizer stock.
D.E.L received research support from BTG International, Metro International Biotech LLC, Renibus Therapeutics, Inc., and Alexion Pharmaceuticals.
